# Angiomatoid Fibrous Histiocytoma: A Tumor With Uncertain Behavior and Various Clinicopathological Presentations

**DOI:** 10.7759/cureus.28985

**Published:** 2022-09-09

**Authors:** Hina Maqbool, Shaarif Bashir, Usman Hassan, Mudassar Hussain, Sajid Mushtaq, Sheeba Ishtiaq

**Affiliations:** 1 Histopathology, Shaukat Khanum Memorial Cancer Hospital and Research Centre, Lahore, PAK; 2 Pathology, Shaukat Khanum Memorial Cancer Hospital and Research Centre, Lahore, PAK; 3 Pathology, Gulab Devi Teaching Hospital, Lahore, PAK

**Keywords:** angiomatoid fibrous histiocytoma, histopathology, neoplasm, angiomatoid fibrous histiocytoma (afh), soft tissue pathology, pathology

## Abstract

Background

Angiomatoid fibrous histiocytoma (AFH) is a rare soft tissue neoplasm of uncertain differentiation, which has various clinical and morphological presentations. Although it behaves in a benign manner, it has malignant potential.

Aim

To share various histological patterns and survival data in our population of this rare entity.

Materials and methods

We studied 25 patients who reported AFH from January 2011 to December 2021. Clinical information, gross and histological features, immunohistochemical results, and survival data were compiled and analyzed.

Results

Among 25 cases reported as AFH, the majority (68%) were males with a mean age of 31.8 years at the time of diagnosis. The most common location was the lower extremity, especially the thigh (56%), and the mean size of the lesion was 55 mm. Most of the lesions were superficial (84%). Grossly, the majority of lesions (76%) had a solid appearance. Microscopically, classic spindle cell morphology was the most common (76%) with a lymphoid cuff and intralesional hemorrhage. Mild cellular atypia was seen in most (92%) of the cases, while some biopsies (8%) had a high-grade morphology. The majority of patients were alive, while one patient died of the disease.

Conclusion

AFH is an under-recognized entity with various clinical and histological presentations and a low malignant potential.

## Introduction

Angiomatoid fibrous histiocytoma (AFH) is a rare soft tissue neoplasm that was first described as “angiomatoid malignant fibrous histiocytoma” by Enzinger in 1979 [1]. However, the subsequent data suggested that it was not a malignant entity and belonged to the category of tumors with uncertain histogenesis and intermediate biological behavior [2]. In the 2020 World Health Organization (WHO) classification of soft tissues, this tumor has been placed under the category of “tumors of uncertain differentiation” [3].

AFH mostly presents as a painless subcutaneous soft tissue mass in the extremities of children and young adults. It has a wide age range with a peak incidence in the first two decades and a mean age of 13 years [1,2,4]. Histologically, the tumors are relatively circumscribed and have a multinodular to lobulated architecture. There are sheets and short fascicles of the oval to spindle cells with bland vesicular nuclei surrounded by dense lymphoplasmacytic infiltrate. Mitoses are sparse and cellular atypia is also infrequently encountered. Intralesional hemorrhage is also a common finding [4,5]. Immunohistochemically, cells are positive for desmin while negative for MyoD1 and myogenin. CD99 and CD68 are variably expressed and the Ki-67 proliferation index is usually low (2-4%) [5,6].

The behavior of AFH is indolent and surgery is the main mode of treatment and is generally curative [7]. Almost 15% of tumors show recurrence and less than 1% present with distant metastasis [8,9].

As AFH is a rare entity with various clinical and morphological presentations, multiple differential diagnoses, and very limited literature, it can be easily missed. Through this article, the authors want to share their experiences to enhance the understanding of clinicians and pathologists in diagnosing and treating cases of AFH.

## Materials and methods

Approval from the Institutional Review Board (IRB number: EX-14-10-21-01) was obtained prior to the commencement of this study in 2021. The hospital information system (HIS) of the Shaukat Khanum Memorial Cancer Hospital and Research Centre was used to retrieve the verified cases of AFH, from the year 2011 to 2021. A total of 25 cases (which fulfilled the inclusion criteria, as mentioned below) were retrieved. The diagnostic material was a combination of resection specimens as well as referred blocks/slides from other hospitals.

Cases with complete demographic and clinical information along with complete diagnostic material and a confirmed diagnosis of AFH were included. Cases with incomplete information or unclear diagnosis were excluded from the study.

Clinical information was acquired from the electronic medical records of the HIS and through telephone calls. The following parameters were recorded: gender and age of the patients, site of the lesion, type of treatment, and current disease status.

Hematoxylin and eosin stained slides of all 25 patients were retrieved from the archives. All cases were re-examined by a specialist pathologist dealing with soft tissue lesions. The gross findings that were recorded from the pathological reports included location, gross morphology, and size of the tumor. On microscopic examination, the dominant histological pattern, presence of intralesional hemorrhage, nature of lymphoid cuff, presence of atypia, mitotic activity, or any peculiar features were noted. Results of immunohistochemical stains including desmin, myogenin, Myo-D1, CD34, etc. were also recorded in relevant cases.

For standardization purposes, intralesional inflammation, lymphoid cuff, nuclear atypia, and fibrosis were categorized as mild, moderate, and marked.

Follow-up data regarding the mode of treatment, disease recurrence, and survival data were acquired via telephonic calls to patients and electronic medical records.

## Results

A total of 25 cases were reported as AFH during the study period of 10 years. Seventeen (68%) patients were males and eight (32%) were females. The mean age at the time of diagnosis was 31.8 years. The most common location was in the lower extremity, especially the thigh (56%), followed by the neck (12%) and axilla (8%). The mean size of the lesion was 55 mm and most of the lesions were superficial, located in the subcutaneous tissue (n = 21, 84%). Grossly, 19 out of 25 (76%) lesions had a solid appearance while the rest six (24%) were either cystic or had both solid and cystic consistency (Table 1).

**Table 1:** Summary of clinicopathological findings in 25 cases of AFH AFH = angiomatoid fibrous histiocytoma; M = male; F = female; HPF = high power field.

Parameters	Value
Mean age	31.8 years
Gender	M = 17, F = 8
Location of lesion	Leg = 14
	Neck = 3
	Axilla = 2
	Arm = 2
	Face = 1
	Chest = 1
	Scapula = 1
	Abdomen = 1
Mean size (mm)	55
Depth	Superficial = 21
	Deep = 4
Gross appearance	Solid = 19
	Solid + cystic = 6
Histology	Spindle = 19
	Epithelioid = 5
Mitotic activity/10 HPF	0-5 = 21
	>5 = 4
Atypia	Mild = 23
	Moderate = 1
	Marked = 1
Mean follow-up time (months)	54
Survival data (n = 15)	Living = 12
	Deceased = 3
	Disease-related mortality = 1

Microscopically, classic spindle cell morphology of the tumor cells was most common (76%) followed by epithelioid morphology (20%). However, there was an overlap of these two morphologies in 12% of the cases. A peculiar single case (4%) with round blue cells was also observed. The lymphoid cuff was present around the lesion in the majority (80%) of the cases with 10 (40%) having a marked, three (12%) moderate, and seven (28%) mild lymphoid cuff. Intralesional hemorrhage was noted in 20 (80%) cases and half of these cases (n = 10) showed both fresh and remote hemorrhage. Mild intralesional inflammation was noted in 14 (56%) of 25 cases while one (4%) had moderate and three cases (12%) had marked intralesional inflammation. Seven (28%) cases did not show any intralesional inflammation. Among the 18 cases that displayed intralesional inflammation, 11 (61%) displayed chronic inflammation composed of lymphoplasmacytic infiltrate. The rest seven (39%) exhibited a mixture of acute and chronic inflammation.

**Figure 1:**
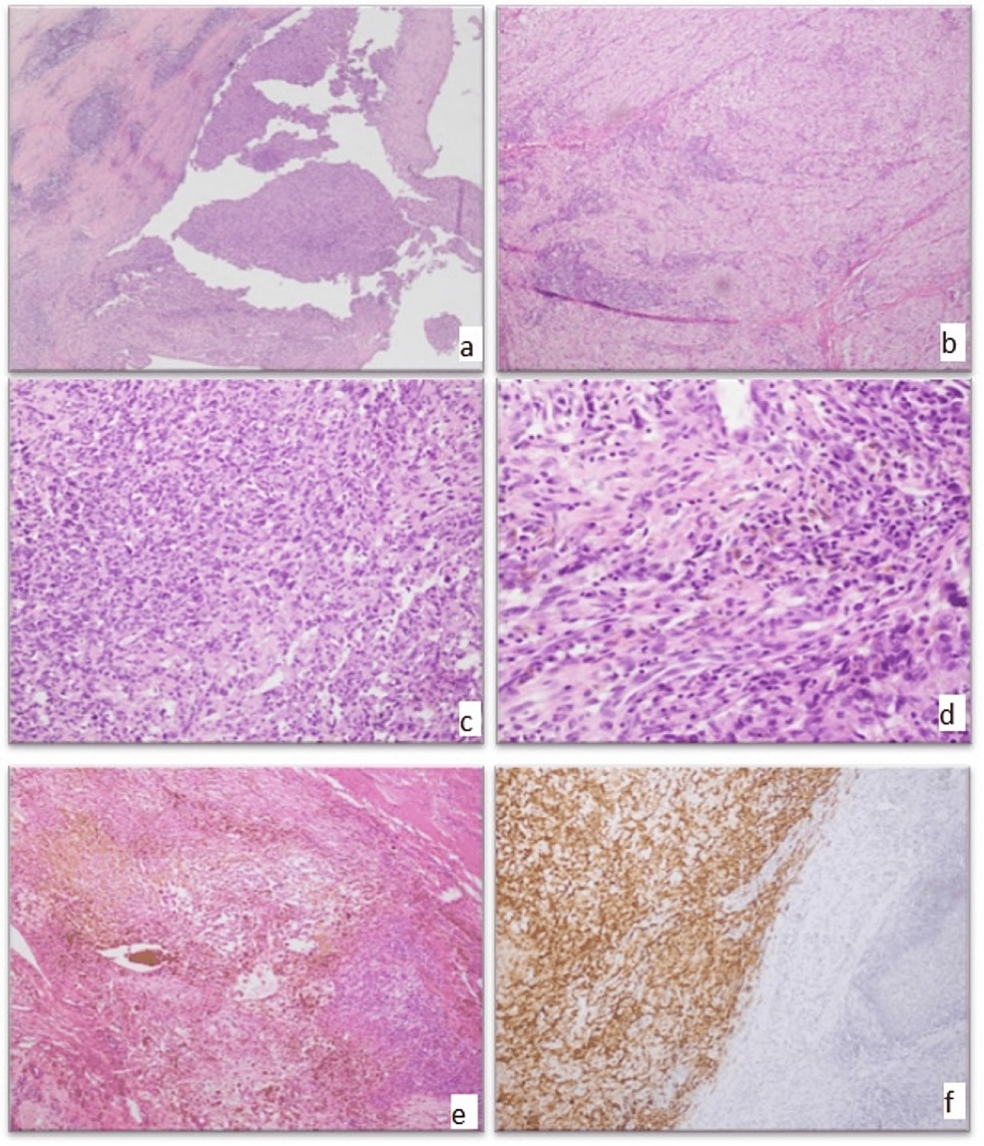
Angiomatoid fibrous histiocytoma classic morphology (A-D) The cystic and solid proliferation of oval to spindle cells with mild atypia, perilesional lymphoid follicles, and intralesional inflammation. (E) Areas of remote and fresh hemorrhage. (F) Desmin immunostain (20x).

Most of the cases (n = 21, 84%) had absent to occasional mitotic figures (0-1). Four cases (16%) revealed increased mitotic activity (>5 mitosis/10 high power field (HPF)) and two (8%) of those four cases had atypical mitosis as well. Mild to moderate fibrosis was observed in 22 (88%) cases. Six of 25 (24%) cases had scattered osteoclast-like giant cells. Mild cellular atypia was seen in 23 of 25 (92%) of the cases while one biopsy (4%) had moderate and one case (4%) had marked atypia. One (4%) case displayed an extensive myxoid change in the stroma while another case (4%) had round blue cell morphology.

Desmin immunohistochemical stain was performed in 18 cases and was positive in eight (44%) cases. SOX10 was performed in 15 cases and only a single case showed focal positivity. CD68 was positive in two of three (67%) cases. CD34 was performed in 12 cases and was negative in all of them. Other stains that were seldomly used included cytokeratin (one case), S100 (five cases), and smooth muscle actin (SMA) (two cases), and were consistently negative.

Follow-up/survival data were available for 15 (60%) cases with a mean follow-up time of 54 months. This included a 10-year follow-up of one of those 15 (6.7%) cases, and a five-year and three-year follow-up of seven (46.7%) and two (13.3%) cases, respectively. The remaining five (33.3%) cases were reported in the last two years. Twelve of the 15 (80%) patients were alive while three (20%) had passed away. Only one of three patients had died of disease while the other two patients died of natural causes, i.e., myocardial infarction and sepsis, respectively. The primary mode of treatment was surgery in all (100%) of the cases with one (6.7%) patient having a history of neo-adjuvant chemotherapy and two (13.3%) having adjuvant therapy (one radiation and one chemotherapy). There were no recurrences and one (6.7%) patient (who died of disease) had lung metastasis (Table 1).

## Discussion

**Figure 2:**
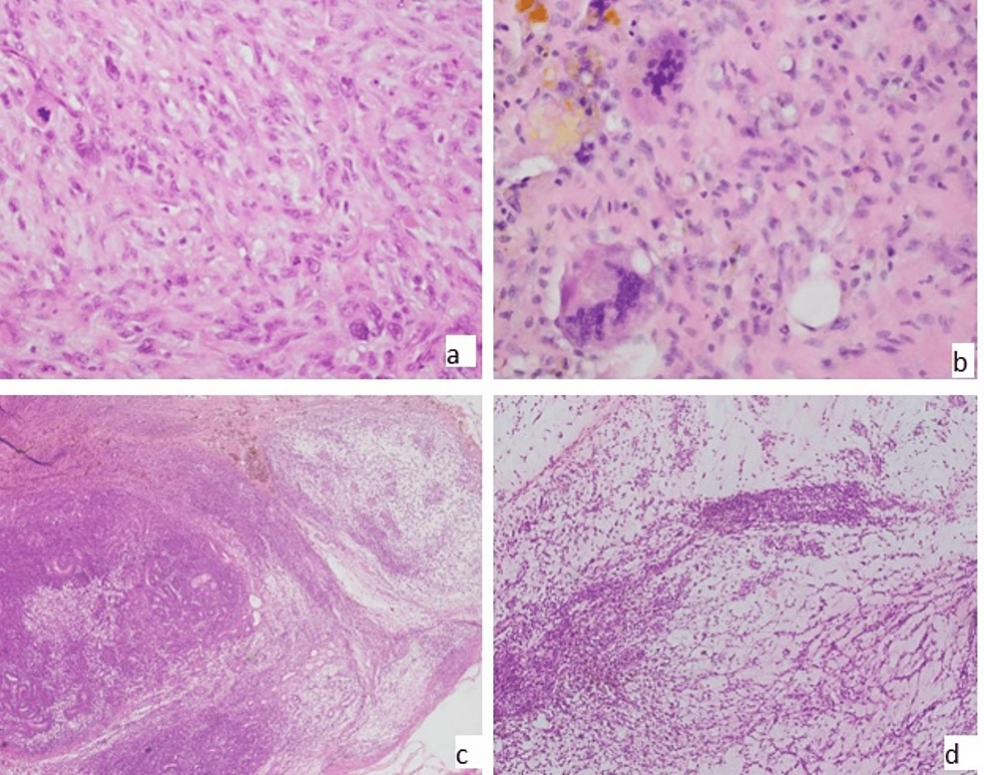
Morphological variations in angiomatoid fibrous histiocytoma (A) Atypical mitosis (20x). (B) Giant cells (20x). (C and D) Myxoid stroma (10x).

Classified under “tumors of uncertain differentiation,” AFH is one of the unique soft tissue tumors with a relatively good prognosis. It usually presents in young adults as a slow-growing, subcutaneous painless soft tissue mass with a predilection toward lower extremities [4,5]. Radiologically, it shows homogeneously hypointense lesions on the T1-weighted image (WI) and heterogeneously hyperintense lesions on the T2-WI [10]. Grossly, the lesions are multinodular, firm, and multicystic with grey to white cut surfaces having areas of hemorrhage [5]. The median size is 2.5 cm but can reach up to 10 cm [1]. Histologically, the tumors are circumscribed and have a multinodular to lobulated architecture with a thick fibrous pseudo-capsule [5]. There are sheets and short fascicles of the oval to spindle cells with bland vesicular nuclei surrounded by dense lymphoplasmacytic infiltrate sometimes forming germinal centers as well [11]. Mitosis is infrequent and cellular atypia can be present [11,12]. Intralesional hemorrhage with blood-filled spaces (pseudoangiomatoid spaces) is also a common finding (Figure 1) [4]. The stroma can sometimes show a myxoid change as well [13]. Giant cells are also present in some cases (Figure 2) [14].

No definite immunohistochemical panel is specific for the diagnosis of AFH. Almost half of the cases are positive for desmin. There can be an expression of SMA and caldesmon. However, Myo-D1, myogenin, and keratins are consistently negative [3,5,15]. CD68 and CD99 can be positive in a subset of the cases presenting a potential diagnostic challenge especially in differentiating from Ewing’s sarcoma [16]. At molecular level, AFH is associated with three characteristic translocations: t(2:22)(q33:q12) (forming the EWSR1-CREB1 fusion gene) [17,18], t(12:22)-(q13:q12) (forming the EWSR1-ATF1 fusion gene) [18-20], and t(12:16)(q13:p11) (resulting in the FUS-ATF1 fusion gene) [21,22]. However, no molecular studies were done in our study.

The epidemiological data of our study were in accordance with other relevant studies. In a similar study of 21 cases of AFH by Shi et al., 10 patients were males and 11 were females and patients' age ranged from eight to 83 years old with a mean age of 26.9 years [23]. Similarly, Tanas et al. concluded that the majority of the subjects were females (seven males and 10 females) with an age range of one to 47 years (mean: 22 years) [16]. About 68% of patients in our study were males while the rest (32%) were females. Age at the time of diagnosis ranged from seven to 65 years with a mean age of 31.8 years. So there was a slight male predilection in our study in contrast to other studies (Table 1).

AFH usually presents as a superficial soft tissue in extremities; however, it can present at any location. A few unusual locations that have been reported in the literature include the brain [24,25], omentum [26], ovary [27], and bone [28,29]. The most common location in our study was the lower extremity (56%) followed by the neck (12%) and axilla (8%). The mean size was 55 mm and most of the lesions were superficial (84%). Most of the cases reported by Shi et al. were also superficial and the most common location was the lower limb (eight of 21) with a mean tumor size of 30 mm [23]. In another study by Alzahim et al., the lower limb was the common location (67%), and the mean tumor size was 40 mm [30].

On gross examination, AFH has solid to cystic multinodular firm lesions with grey-white cut surface and areas of hemorrhage [1]. Most of the cases in our study (76%) had a solid appearance while the rest (24%) were either cystic or had both solid and cystic consistency. This was similar to other studies like Shi et al. in which most of the cases were circumscribed and had a firm consistency [23]. Microscopically, the tumors were circumscribed and lobulated with a thick fibrous pseudo-capsule. A dense lymphoid cuff is mostly present in the surroundings. Cytologically, the cells are oval to spindle-shaped arranged in sheets and fascicles. Usually, there is no marked atypia or significant mitotic activity. Intralesional hemorrhage with pseudoangiomatoid space formation is also a common finding [5,11,12,23].

Apart from the classic histology, some variant/peculiar findings have also been reported in the literature. Kao et al. reported 13 cases of AFH in which two cases displayed giant cells and another two cases had marked nuclear atypia. Furthermore, three cases showed dense sclerosis and another three cases had a myxoid stroma [14]. Chen et al. reported the presence of clear cells, rhabdomyoblast-like cells, and cords of tumor cells in a myxoid stroma [27]. Bohman et al. reported a total of 27 cases of AFH. Ten cases displayed significant areas of sclerosis, nine displayed at least moderate pleomor­phism, and eight had scattered eosinophils in the stroma [11]. A myxoid variant was also reported by Schaefer et al. in a series of 21 cases [13].

**Figure 3:**
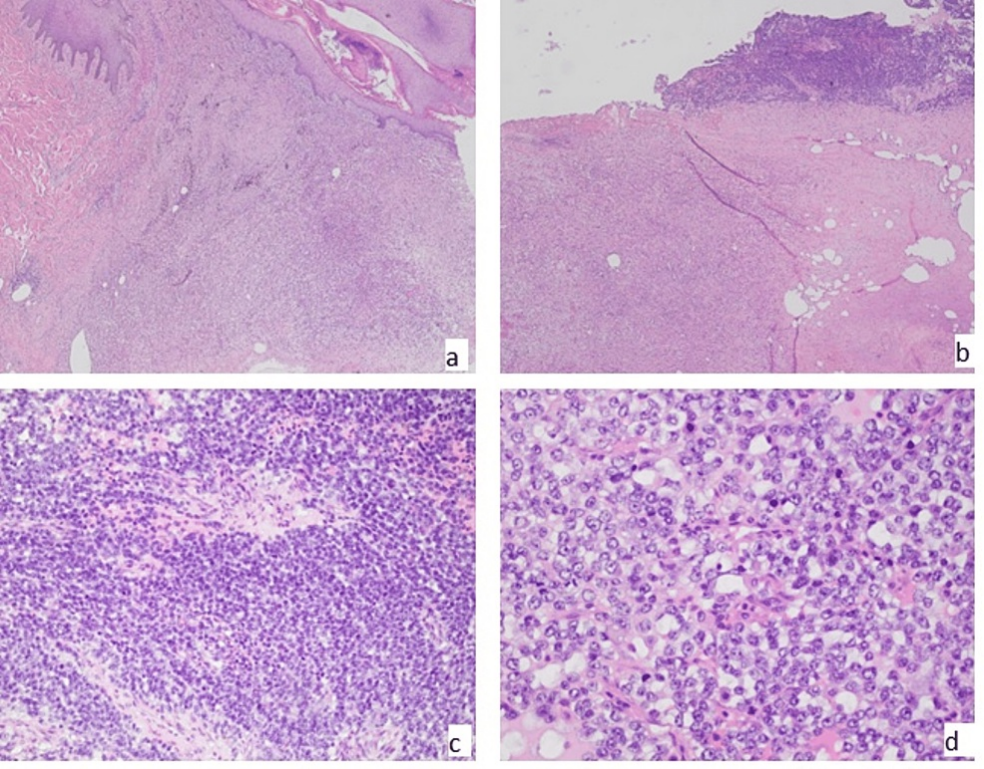
Angiomatoid fibrous histiocytoma (AFH) with high-grade transformation to round blue cell sarcoma (A) Superficial areas of classic morphology (4x). (B) Interface between classic and high-grade areas (4x). (C and D) Round blue cell sarcoma arising in AFH with atypical round to oval cells with vesicular nuclei and brisk mitotic activity (20x and 40x).

In our study of 25 cases, spindle cell morphology was most common (76%) with surrounding moderate to marked lymphoid cuff in more than half of the cases. Intralesional hemorrhage with pseudoangiomatoid spaces was noted in 20 (80%) of the cases. Intralesional inflammation was noted in 18 (72%) cases with 11 of those 18 (61%) showing chronic lymphoplasmacytic inflammation (Table 1). Peculiar morphological findings in our study included myxoid stroma in one (4%) case, osteoclast-like giant cells in six (24%), intralesional adipose tissue in one (4%), round blue cell morphology in one (4%) (Figure 3), high-grade atypia in one (4%), increased mitotic activity (>5 mitosis/10 HPF) in four (16%), and atypical mitosis in two (8%) of the 25 cases (Figure 2). In the cases with atypical morphology, extensive sampling was performed and areas of classic morphology were identified, which helped in the final diagnosis.

Immunohistochemistry has a limited role in the diagnosis of AFH as it lacks a specific immune profile. Positivity for desmin, vimentin, and CD68 is seen in most of the cases. Shi et al. reported vimentin positivity in all 21 (100%) cases, epithelial membrane antigen (EMA) in 11/21 (52%), desmin in 14/21 (66%), and CD68 in 17/21 (88%) cases [23]. In our study, desmin positivity was seen in 44% of the cases, CD68 in 67%, and SOX10 in 6.7% of the cases. These non-specific markers pose a diagnostic dilemma to the pathologists, especially when dealing with a case having a non-conventional morphology, and more often than not, AFH becomes a diagnosis of exclusion. In such cases, fluorescence in situ hybridization (FISH) for EWSR1 gene fusions can be helpful.

**Figure 4:**
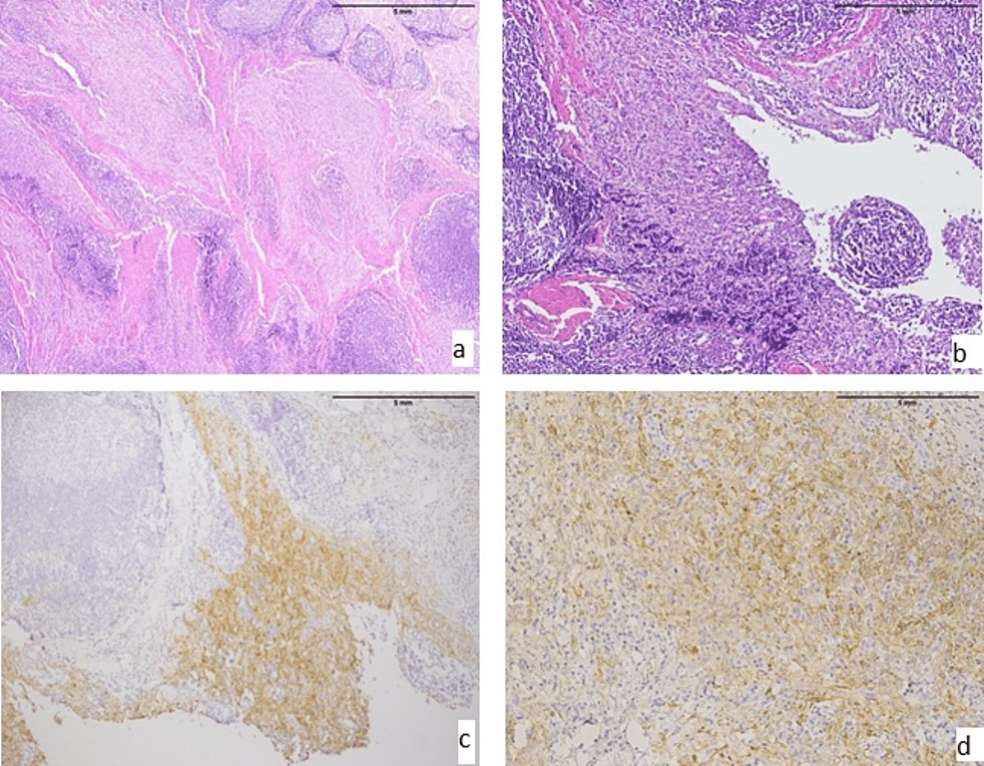
ALK expression in angiomatoid fibrous histiocytoma (AFH) (A and B) Classic histology of AFH with spindle cells in sheets and fascicles with surrounding lymphoid follicles (10x and 20x). (C and D) ALK immunostain with moderate to strong cytoplasmic staining in lesional areas (10x and 20x).

Interestingly, anaplastic lymphoma kinase 1 (ALK1) immunostain was performed as a part of the workup in a single case and it showed diffuse cytoplasmic positivity (Figure 4). In a fairly recent study, Cheah et al. [31] also came across cases of AFH, which were misdiagnosed as inflammatory myofibroblastic tumors (IMT) based on anaplastic lymphoma kinase (ALK) positivity. In their study, nine of 11 cases were positive for ALK1 immunostains. They further investigated their findings by performing FISH for ALK1 gene rearrangement and EWSR1 gene fusions. Almost all cases showed EWSR1 gene fusions and none showed ALK1 gene rearrangement. The mechanism of this unusual ALK immunohistochemical positivity without any molecular basis still remains a mystery and warrants further investigation, as, in the future, ALK inhibitors such as crizotinib might be a potential player in the treatment of AFH.

Surgical excision is the main mode of treatment in AFH and has proven to be curative in most of the reported cases. However, recurrence and metastasis have been reported. Enzinger et al. reported that 63% had local recurrence and 21% had metastasis, and 12% of patients died of the disease [1]. Pettinato et al. [32] reported recurrence occurred in 25% of cases, metastasis in 5%, and death in 5% of the cases. No recurrence or metastasis was reported by Alzahim et al. in their case series [30]. In a series by Shi et al., only 2/21 (9.5%) patients had local recurrence at three and six months after resection [23]. In our study, follow-up information was available for 15 of 25 (60%) cases with a 10-year follow-up of one of those 15 (6.7%) cases, a five-year follow-up of seven (46.7%), and a three-year follow-up of two (13.3%) cases. The primary mode of treatment was surgery in all 15 (100%) cases with one (6.7%) patient having a history of neo-adjuvant chemotherapy and two (13.3%) having adjuvant therapy (one radiation and one chemotherapy). No added benefit of adjuvant or neo-adjuvant chemo-radiation was proven. Twelve of 15 (80%) patients were alive and only one (6.7%) patient had disease-related death. The patient who died of the disease received adjuvant chemotherapy and had metastasis to the lung. No recurrence of the disease was reported in our patient pool.

The differential diagnosis for AFH includes IMT, aneurysmal fibrous histiocytoma, spindle cell hemangioma, and metastatic tumor deposits within lymph nodes. Compared to AFH, the tumor cells of IMT are myofibroblasts, have distinct cell borders and eosinophilic cytoplasm, and the inflammatory cells are intimately intermingled with the neoplastic cells without forming a peritumoral rim. Moreover, there is positivity for desmin, SMA, and ALK in IMT. Aneurysmal fibrous histiocytoma (which is an uncommon variant of dermatofibroma) has blood spaces and is superficially located just like AFH and can cause diagnostic confusion. However, in addition to the features of a typical dermatofibroma, it has a more heterogeneous cell population than AFH (often containing giant cells and siderophages), has large cleft-like or cavernous blood-filled spaces, lacks a surrounding lymphoplasmacytic infiltrate, and is also desmin negative. Spindle cell hemangioma usually occurs on extremities, mostly in the dermis and subcutis, and has cavernous vascular spaces. But unlike AFH, it is poorly circumscribed and has true vascular spaces lined by attenuated endothelial cells. Metastatic nodal deposits in the lymph nodes show the surrounding true nodal architecture, which includes sub-capsular and medullary sinuses, in contrast to the randomly distributed germinal centers, which are seen in AFH.

Overall, the authors believe that AFH is an under-recognized entity as it can have various clinical and histological patterns. In this study, for example, AFH was found at different locations, there was male dominance (unlike in previous studies), and various gross and histological presentations were recorded. But even in those histologically divergent cases, some areas of classic morphology were present (after further extensive sampling), which helped in the diagnosis of AFH. A peculiar case of round cell sarcoma arising in AFH was also encountered in our study. The only limitation of our study was the lack of molecular data for EWSR gene rearrangements due to financial constraints; however, only the cases with the classic clinical and pathological presentation were included in the study to avoid any confusion. This study provides a detailed insight to help pathologists in their diagnostic approach toward this benign entity with malignant potential.

## Conclusions

AFH is a rare but well-reported soft tissue neoplasm with specific histological and non-specific immunohistochemical features. For an accurate diagnosis, it is important to perform extensive sampling and look for classic morphological areas, and exclude other differentials. Moreover, FISH for EWSR1 gene fusions can also be of help. The prognosis is generally good and surgical excision is usually curative.
